# Social Media under the Skin: Facebook Use after Acute Stress Impairs Cortisol Recovery

**DOI:** 10.3389/fpsyg.2017.01609

**Published:** 2017-09-19

**Authors:** Holly M. Rus, Jitske Tiemensma

**Affiliations:** Psychoneuroendocrinology Lab, Psychological Sciences, University of California, Merced Merced, CA, United States

**Keywords:** stress, cortisol, TSST, Facebook, social media use, well-being

## Abstract

Social media's influence on stress remains largely unknown. Conflicting research suggests that Facebook use may both enhance *and* undermine psychosocial constructs related to well-being. Using novel experimental methods, this study examined the impact of social media use on stress recovery. Facebook users (*n* = 92, 49 males, mean age 19.55 *SD* = 1.63) were randomly assigned to use their own Facebook profile or quietly read after experiencing an acute social stressor. All participants showed significant changes in subjective and physiological stress markers during recovery. Participants who used Facebook experienced greater sustained cortisol concentration (*p* < 0.05) when controlling for gender and emotional investment in the website compared to controls. Results suggest that social media use may delay or impair recovery after experiencing an acute social stressor. This novel study incorporated objective physiological markers with subjective psychosocial measures to show that Facebook use may negatively impact well-being.

## Introduction

To date, Facebook remains the most popular social networking site, with over 1 billion worldwide users (Facebook Company Information, 2016) and 71% of online U.S. adults naming it as their preferred platform (Duggan et al., 2015). Use of the site remains as varied as it does popular. Approximately 44% of U.S. adults now report receiving their news from Facebook (Gottfried and Shearer, 2016), while research has also found that the site serves a starkly contrasted, but still valuable, utility in coping with campus violence (Vicary and Fraley, 2010).

Several studies have examined potential associations between Facebook use and outcomes related to psychosocial well-being, many of which have provided mixed and sometimes conflicting results. For example, use has been associated with increased self-esteem (Best et al., 2014), general well-being (Kim and Lee, 2011), enhanced social support (Bender et al., 2011; Liu and Yu, 2013; Troncone et al., 2015), and overall life satisfaction (Valenzuela et al., 2009; Nabi et al., 2013); just as well as with greater distress (Chen and Lee, 2013), induction of negative social comparison (Chou and Edge, 2012), and declines in subjective well-being (Kross et al., 2013; Verduyn et al., 2015). One of the first studies to examine Facebook use and health found an association between online social integration and reduced mortality rate (Hobbs et al., 2016); however, underlying mechanisms were not explored. Research has also shown that gender may play an important role in how Facebook influences well-being. Not only are females more likely to use Facebook (Anderson, 2015), they may be more susceptible to feeling threatened by specific information displayed on the site (e.g., McAndrew and Shah, 2013).

The link between Facebook use and stress receives substantial public attention, and is often touted as a causal relationship (e.g., Heid, 2017). However, this relationship has not been experimentally investigated. To date, one of the few studies to objectively measure stress in relation to Facebook use found that adolescents with larger Facebook networks showed greater cortisol release compared to those who spent less time interacting with Facebook peers (Morin-Major et al., 2016).

As an objective marker of the body's physiological stress response (Granger et al., 1999, 2007; Hellhammer et al., 2009; Nater and Rohleder, 2009; Birkett, 2011), cortisol can expand our understanding of the impact of Facebook use on stress. While many studies have focused on mechanisms that may *buffer* acute laboratory stress (e.g., Creswell et al., 2005; Arch et al., 2014; Inagaki and Eisenberger, 2016), fewer have looked at what may promote physiological recovery.

Considering both the negative consequences of stress (e.g., Herbert and Cohen, 1994) and the widespread adoption of Facebook and its abundant presence in the daily lives of many, we aimed to test if social media can truly *get under the skin* by influencing recovery from stress. We hypothesized that should social media delay physiological and psychosocial stress recovery, participants who used Facebook immediately following an acute social stressor would show elevated and sustained salivary cortisol output, as well as subjective stress. We also explored if users highly invested in Facebook may differ in recovery, and if this effect may relate to gender.

## Materials and methods

### Participants

Facebook users (*n* = 112 undergraduates) were recruited from a campus-wide participant pool system. All participants had an active Facebook account and were given course credit in exchange for participation. Self-reported medical diagnoses (e.g., anxiety, PTSD) and use of substances known to influence HPA-axis activity (e.g., steroids, hormonal contraceptives) were considered as exclusion criteria. This study was carried out in accordance with the recommendations of Expedited Review, The University of California, Merced Institutional Review Board, with written informed consent from all subjects. All subjects gave written informed consent in accordance with the declaration of Helsinki. The protocol was approved by the University of California, Merced Institutional Review Board. All data collection complied with current APA ethical standards.

### Experimental tools

#### Stress induction and physiological measures

The Trier Social Stress Test (TSST; Kirschbaum et al., 1993) has been shown to reliably induce acute stress in the majority of participants in numerous studies (Birkett, 2011). Specifically, it is known to induce a threat to social esteem and reliably induces an increase in cortisol and in negative self-related cognitions and emotions (Dickerson and Kemeny, 2004; Dickerson et al., 2004). In the current study, ~20 min after arriving in the lab, participants were instructed to spend 5 min preparing a speech that could be used in an interview for their ideal job. They then spent 5 min performing the speech in front of a disapproving committee of three presumed experts in a small laboratory room. Participants then counted backward from 1,687 by intervals of 13 for 3 min. When mistakes were made, participants were told to begin again. To further induce stress, participants were video and audio recorded during the speech and math tasks. In addition, committee members wore white lab coats and carried clipboards to enhance the illusion of being experts. The committee always consisted of mixed-gender, undergraduate members (i.e., two males and one female, or two females and one male) who were present in the laboratory room only for the duration of the stress task.

##### Saliva samples

To control for natural cortisol fluctuations during the day (Kirschbaum and Hellhammer, 1989; Schultheiss and Stanton, 2009), all data were collected between 1:00 and 5:00 p.m. (i.e., each participant arrived for their 90-min laboratory session at either 1:00 or 3:00 p.m.). To ensure quality of saliva samples and to avoid temporary elevation of cortisol levels (Schultheiss and Stanton, 2009), participants were instructed to refrain from eating, smoking, consuming caffeine, drinking beverages other than water, brushing their teeth, or vigorously exercising in the 30 min before arriving for the study^1^. All samples were collected using salivette collection tubes (Sarstedt Co., Nümbretch, Germany). Participants placed a cotton roll under their tongue for 2 min of collection. To account for the natural fluctuation of cortisol in reaction to acute stress (Engert et al., 2011), saliva was collected at baseline, and at 8, 20, and 45 min post-stressor onset. Cortisol samples were immediately frozen and immunoassayed on site at a later date. All samples were placed in a –20°C freezer. Thawed samples were centrifuged and assayed in duplicate with a test volume of 25 μL. A commercially available enzyme immunoassay kit was used without modifications to the manufacturer's recommended protocol (Salimetrics, State College, PA). Sensitivity ranged from 0.007 to 3.0 μg/dL. Intra-assay and inter-assay coefficients of variation were less than 15%.

##### Blood pressure and heart rate

Blood pressure and heart rate were simultaneously measured with an Omron 10 Series digital blood pressure monitor cuff placed around the non-dominant upper arm at baseline, 8, 20, and 45 min post-stressor onset.

#### Psychosocial measures

##### Facebook use

The Facebook Intensity Scale (FBI) measures emotional connectedness to the site and integration of site use into the lives of users (Ellison et al., 2007). The 9-item scale asks participants to rate statements such as, “Facebook has become part of my daily routine,” on a five-point scale from 1 (strongly disagree) to 5 (strongly agree). The scale also measures number of Facebook friends as well as average daily time spent actively using Facebook over the past week. Intensity score is computed by averaging all items in the scale, with higher scores indicating higher intensity. Scale validity has not been established; however, the current sample showed moderate reliability (Cronbach's α = 0.77).

The Facebook Activity Survey (Junco, 2012) measures frequency of specific activities within Facebook. Examples include frequency of posting status updates, sharing links, and sending private messages on a scale of 1 (never) to 5 (very frequently, 100% of the time). All participants reported their normal Facebook use habits at baseline. Experimental participants completed an adapted version of the survey regarding their specific use of the site during 30 min of recovery. In both cases, frequency of each activity was averaged across participants with higher scores indicating more frequent activity.

Participants were also asked which method they most commonly used to access Facebook (i.e., mobile app, website from a computer, or both). In addition, participants in the Facebook use condition were asked how using Facebook for 30 min in one sitting compared to their normal use (i.e., they normally use it less, the same, or more), if they did anything during these 30 min that they normally would not do, and if so, what they did.

All questionnaire items assessing Facebook use and stress were asked during follow-up (i.e., *after* participants had undergone both the acute stressor and used Facebook if they were in the experimental condition). This was done in effort not to bias participants toward the study's true purpose.

Participants identified under which state they were most likely to use Facebook (lonely, bored, stressed, sad, or anxious) by rating their agreement on a 1 (strongly disagree) to 5 (strongly agree) scale for the item, “I find myself wanting to use Facebook most when feeling X” for each state. In addition, participants responded to the following statement: “Please rate how stressed using Facebook makes you feel in general,” on a five-point scale ranging from 1 (not at all) to 5 (extremely). Participants also rated the following statements on five-point scales ranging from 1 (strongly disagree) to 5 (strongly agree): (1) “In general, I like to use Facebook when I am stressed,” (2) “In general, using Facebook when I am stressed makes me feel *less* stressed,” and (3) “In general, using Facebook when I am stressed makes me feel *more* stressed.” Participants in the Facebook use condition were asked to select which statement they agreed with most after using Facebook for 30 min: (1) “Using Facebook made me feel *less* stressed,” (2) “Using Facebook made me feel *more* stressed,” or (3) “Using Facebook did not change my stress level.”

##### Mood

The Positive and Negative Affect Schedule (PANAS; Watson et al., 1988) measured change in mood from baseline to follow-up. The 20-item scale consists of words describing 10 negative emotions and 10 positive emotions. Participants indicated on a scale of 1 (very slightly or not at all) to 5 (extremely) how they felt in the present moment for each emotion. Higher scores for each emotion indicated higher levels of positive or negative affect respectively. The well-validated scale (Crawford and Henry, 2004) showed high internal consistency for baseline ratings of positive affect (Cronbach's α = 0.89) and negative affect (Cronbach's α = 0.79), and for 45-min post-stressor onset ratings of positive affect (Cronbach's α = 0.91) and negative affect (Cronbach's α = 0.83).

In addition, mood was directly assessed after recovery for those in the Facebook use condition with the following item: “Please indicate which statement you agree with most: (1) Using Facebook increased my positive mood, (2) Using Facebook increased my negative mood, or (3) Using Facebook did not change my mood.”

##### Well-being

**Figure 1:**
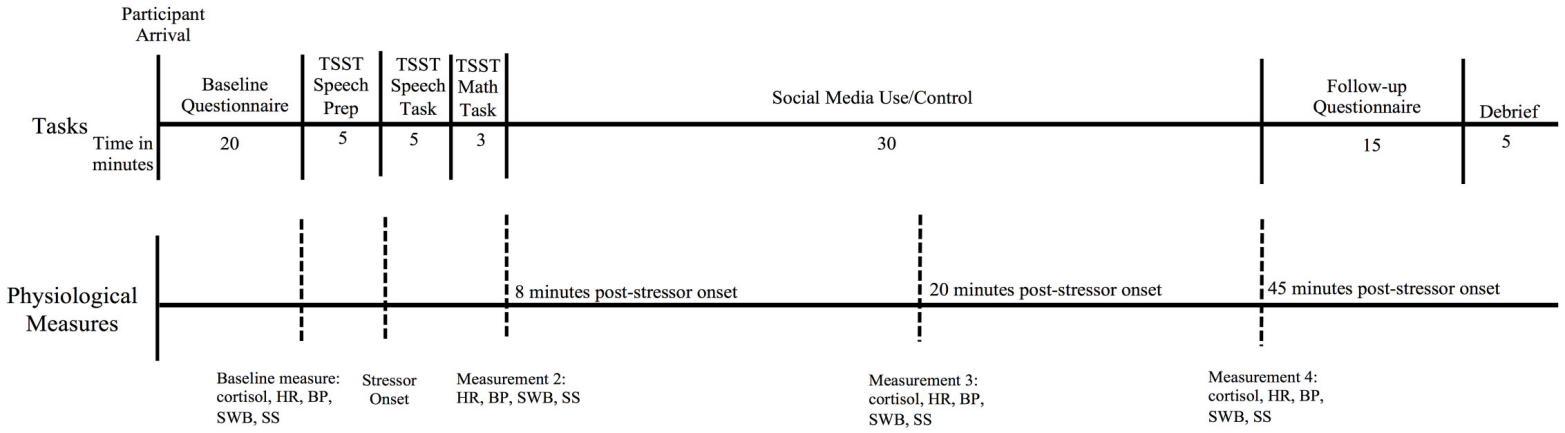
Timeline for procedural tasks and physiological sample measurements. TSST, Trier Social Stress Test; HR, heart rate; BP, blood pressure; SWB, subjective well-being; SS, subjective stress.

Subjective well-being was assessed at each saliva sample collection time point (see Procedure and Figure 1) with a visual analog scale anchored at “not well” and “extremely well” for the statement, “What is your overall sense of well-being right now?” Participants responded by marking along a 15 cm line. Responses were measured and rounded up to the nearest millimeter, then converted to a 15-point continuous scale with higher scores indicating greater feelings of well-being. In addition, well-being was directly assessed after recovery for those in the Facebook use condition on a scale of 1 (not at all) to 4 (a lot) with the following item: “How much did using Facebook influence your sense of well-being either positively or negatively?”

##### Subjective stress

Subjective stress was assessed at each saliva sample collection time point with present-moment ratings of feeling tense and anxious with the items, “How tense/anxious are you feeling right now?” Each item was rated from “not at all” to “extremely” along the same visual analog scale as the well-being measure. The descriptive terms “tense” and “anxious” were used instead of “stress” for these items in effort not to bias participants to the true purpose of the study.

### Design and procedure

To assess the effect of Facebook use on stress recovery, participants in the current study came into the lab believing they would be taking a survey on their Facebook use habits and providing physiological samples (i.e., saliva, blood pressure, heart rate) to assess well-being. All participants underwent an unexpected, acute social stressor before half were randomly assigned to log into their own Facebook account (experimental condition), and half were given neutral reading materials (control condition) for 30 min of recovery.

Participants completed all procedures in a single, 90-min laboratory session. All procedures took place within the same laboratory room where only the individual participant and experiment leader were present (with the exception of the portion involving the TSST committee). The experiment leader (a female graduate student not involved in the TSST) explained that the study aimed to look at the influence of social media use on well-being. In the description provided by the online participant recruitment system, participants were told they would need to know their Facebook login information in order to participate in the study; however, participants randomly assigned to the Facebook use condition did not know they would be using Facebook during the study until the moment the experiment leader asked them to log into their own account (~35 min into the study). Control participants never used their login information during the study. All participants were unaware that the study involved a stress task beforehand.

After the study was explained and informed consent collected, all participants completed baseline measures. Following, the experiment leader left the room, and the committee entered to conduct the TSST. The experiment leader then returned, excused the committee, and instructed participants on how to proceed. During 30 min of stress recovery, participants randomly assigned to the experimental condition (*n* = 42) logged into their own Facebook account on the same laboratory computer used to complete baseline and follow-up measures (a laptop stored out of sight during the TSST). They were instructed to use Facebook as they wished with the exception of disclosing any information about their current participation in the study. Participants in the control condition remained in the same room with optional reading materials (scientific journals and magazines). The experiment leader remained in the room with each participant during recovery in order to collect physiological samples and subjective stress measures; however, participants were instructed not to speak to the experiment leader during recovery. Salivary cortisol output, heart rate, blood pressure, subjective stress, and subjective well-being were assessed at baseline, and at 8, 20, and 45 min post-stressor onset (see Figure 1 for study timeline). After recovery (45 min post-stressor onset), all participants completed measures of changes in mood and reported the general influence of Facebook use on stress and well-being. In addition, participants in the experimental condition reported their Facebook activity during the recovery period and the immediate influence of Facebook use on stress, well-being, and mood.

After the final saliva measure and follow-up questionnaire, all participants were debriefed about the study's true purpose of testing the effect of social media use on stress recovery.

### Statistical analyses

Analyses were conducted using SPSS Version 24.0 (SPSS Inc., Chicago, IL, USA). Analysis of covariance (ANCOVA) with gender and Facebook Intensity (i.e., emotional connectedness to the site) as covariates was used to test the effect of Facebook use on acute stress recovery. To check the appropriateness of assumptions for the statistical analysis, the Kolmogorov-Smirnov test, histograms, and scatter plots were used. Analyses included mean change scores from baseline to 45 min post-stressor onset for positive and negative affect, mean change scores from 8 to 20 min post-stressor onset for blood pressure and heart rate, and mean change scores from 20 to 45 min post-stressor onset for all other variables. A median split was applied to Facebook Intensity score, creating a dichotomous variable (low, high) for use as a covariate. Seven participants (3 in the Facebook condition, 2 males; 4 in the control condition, 1 male) were missing either the 20- or 45-min saliva sample and thus were not included in final analyses. To account for skewness, cortisol measures were log-transformed before analyses. Unless otherwise noted, effect sizes are presented as partial η^2^, which represents the proportion of explained variance between the predictors and the outcome, with values of 0.01, 0.06, and 0.14 indicating small, medium, and large effect sizes, respectively (Cohen, 1988). Significance was set at *p* ≤ 0.05.

## Results

### Sample characteristics

Given our interest in stress recovery, participants who maintained stable levels or showed a decrease in cortisol output concentration in response to the stress induction (*n* = 10) were excluded from analyses (i.e., they did not experience an increase in physiological stress and therefore did not experience recovery). Participants who reported current use of prescription medication containing cortisol, cortisone, or hydrocortisone were excluded from analyses (*n* = 2). One participant was identified as an extreme outlier for cortisol (i.e., score > 4 *SD*s above the mean) and was excluded from analyses.

The final sample of participants (*n* = 92; 43 females, mean age = 19.74 years, *SD* = 1.51, BMI = 27.31, *SD* = 8.42; and *n* = 49 males; mean age = 19.55, *SD* = 1.63, BMI = 21.56, *SD* = 7.54) identified as being Hispanic/Latino (44.6%), Asian/Pacific Islander (20.7%), biracial (14.1%), Caucasian (10.9%), or African American/Black (4.3%). The majority of the sample (68.5%) identified as first-generation college students.

Average weekly alcohol consumption within normal range was permitted, however the majority of participants (73.9%) reported zero consumption. Three participants reported current use of recreational drugs while one reported current use of tobacco products. Only 15.6% of female participants (*n* = 7; 4 control, 3 Facebook use) reported current use of hormonal contraceptives. Three participants reported current anxiety disorder diagnosis; however, none reported current use of anti-anxiety medication. No participants reported current diagnosis of post-traumatic stress disorder nor current use of anabolic steroids. None of these participants showed extreme scores on any outcome measure, nor did their stress response patterns widely diverge from the rest of the sample. Thus, all were retained in analyses. Seven participants quit the study before or during the acute stress induction (see Design and Procedure).

Independent-samples *t-*tests showed no significant condition differences on any measure of Facebook activity or on any baseline physiological measure (see Table 1). Compared to control participants, participants in the Facebook use condition were more likely to report that using Facebook when stressed makes them feel *less* stressed, *t*_(90)_ = 2.06, *p* = 0.04, 95% CI [−0.81, −0.02], *d* = 0.34. Participants showed no other significant condition differences on any item regarding the general influence of Facebook use on stress and well-being.

**Table 1:** Full sample and condition values for baseline and Facebook use measures.

	**Full sample**	**Control condition**	**FB condition**
*n*	92	50	42
Females (*n*)	43	28	15
Age	19.64 (1.57)	19.88 (1.78)	19.36 (1.25)
**FB ACTIVITY**			
FB friends	<399	<399	<299
Years with FB account	<5	<5	<5
Daily use (minutes)	<44	<44	<44
FBI low intensity (*n*)	39	21	18
FBI high intensity (*n*)	53	29	24
Most common activities:	Liking posts, following links to other websites, viewing photos	Liking posts, following links to other websites, scrolling newsfeed without clicking	Viewing videos, viewing photos, liking posts
**I FIND MYSELF WANTING TO USE**			
**FB MOST WHEN FEELING:**			
Lonely	45%	44%	45%
Bored	92%	94%	90%
Stressed	32%	28%	36%
Sad	18%	18%	19%
Anxious	27%	26%	29%
In general, how stressed does using FB make you feel?	1.38 (0.55)	1.40 (0.57)	1.36 (0.53)
In general, I like to use FB when I'm stressed	2.83 (1.03)	2.90 (0.99)	2.74 (1.08)
In general, using FB when stressed makes me feel *less* stressed	3.32 (0.97)	**3.04 (1.03)**	**3.45 (0.86)**
In general, using FB when stressed makes me feel *more* stressed	2.41 (0.99)	2.44 (0.97)	2.38 (1.04)
**PSYCHOSOCIAL STRESS**			
Tension	3.29 (2.78)	3.02 (2.52)	3.62 (3.07)
Anxiety	3.41 (2.86)	3.61 (2.95)	3.12 (2.76)
Well-being	10.93 (2.72)	11.29 (2.49)	10.48 (2.95)
Positive affect	29.40 (8.24)	28.22 (7.67)	30.80 (8.74)
Negative affect	15.05 (4.98)	14.76 (4.02)	15.40 (5.97)
**PHYSIOLOGICAL STRESS**			
Systolic blood pressure	112.34 (12.38)	112.18 (14.15)	112.55 (10.04)
Diastolic blood pressure	71.77 (7.87)	71.94 (8.18)	71.57 (7.58)
Heart rate	72.10 (10.86)	73.98 (11.19)	69.88 (10.14)
Cortisol	0.17 (0.11)	0.18 (0.12)	0.17 (0.10)

*Reported values reflect n = 92. FB, Facebook. FBI, Facebook Intensity Scale. Participants responded to number of FB Friends, Years with Facebook account, and Daily use as closed-ended questions. For these items, values represent the number, years, and time in minutes that correspond to the median responses from ordinal 1-to-5 scales. FBI low/high intensity represent number of participants in each condition after a median split was applied to the Facebook Intensity Scale. Percentages for each state (lonely, bored, etc.) represent percentage of participants who agreed or strongly agreed with each statement. All other values represent baseline condition means and standard deviations. Cortisol values represent raw salivary cortisol concentration in μg/dL. Bolded values indicate a significant difference between conditions at p ≤ 0.05*.

### Effect of facebook use on psychosocial stress recovery

Fifty-two percent of the participants in the Facebook use condition identified the Facebook mobile app as their most common method of access, while 16.7% reported most commonly using the website on a computer, and 31% reported using both the mobile app and a computer to access Facebook equally. When asked what they did during 30 min of stress recovery, participants in the Facebook use condition identified passively scrolling newsfeed, viewing videos, and following links as the activities they spent the most time doing. The majority of participants (66.7%) indicated that they normally spend less than 30 min using Facebook in one sitting. However, the majority of participants (66.7%) also indicated that using Facebook for 30 min did not cause them to engage in any activities during one sitting in which they normally would not. Participants who reported doing something they normally would not do because of the extended use time (*n* = 14) almost exclusively reported that they watched videos. When asked about the effect of Facebook use on mood, 54.8% of participants reported a positive change in mood, 14.3% reported a negative change in mood, and 31.0% reported no effect on mood during recovery. In addition, 59.5% reported that using Facebook made them feel *less* stressed, 7.1% reported feeling *more* stressed, and 33.3% reported experiencing no change in stress level as a result of using Facebook during recovery. Seventy-nine percent of participants reported that using Facebook changed their sense of well-being during recovery.

**Figure 2:**
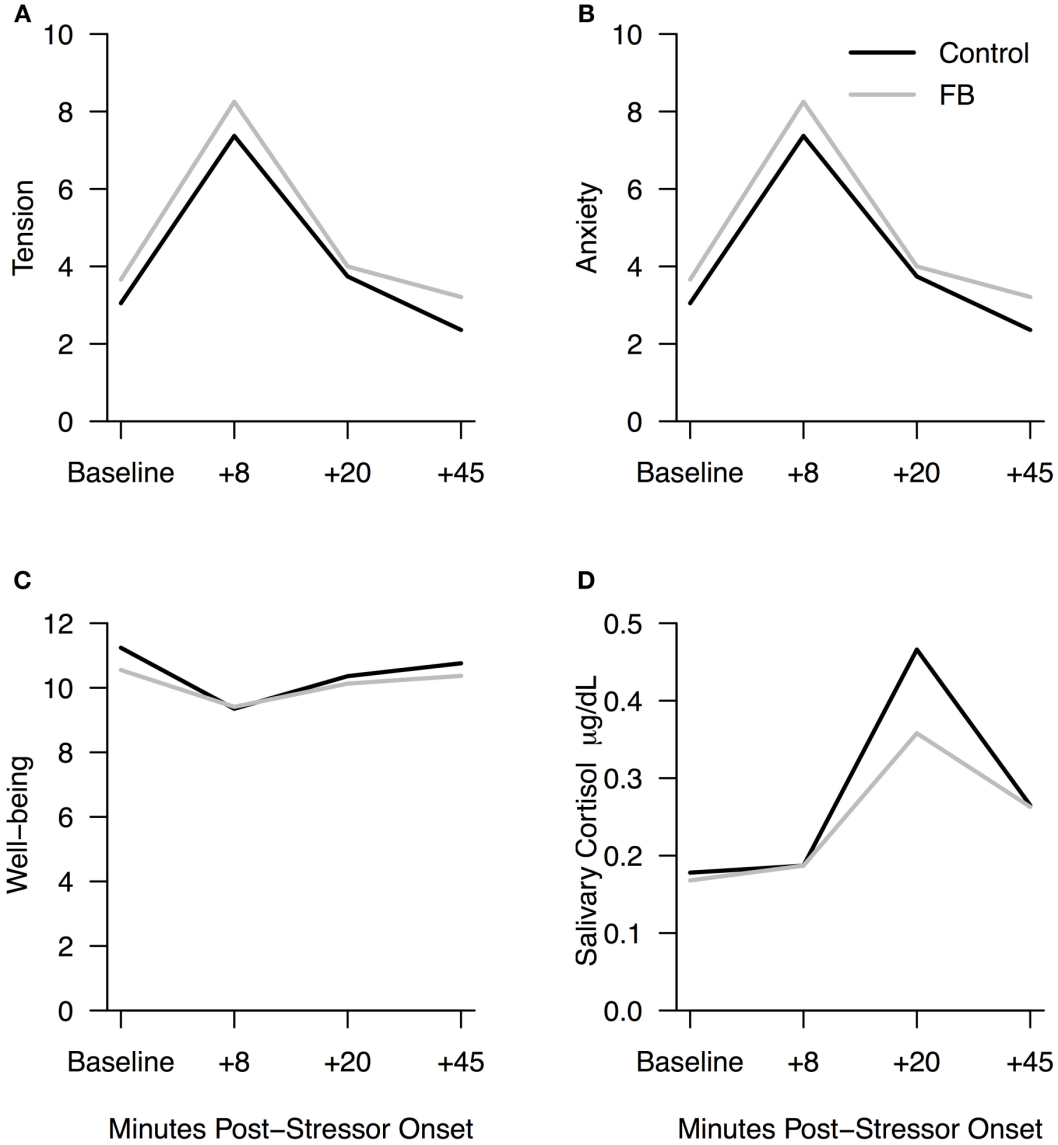
**(A–D)** Subjective stress markers and cortisol for Facebook and Control conditions. Facebook and Control conditions showed no significant differences at any time point (*p* < 0.05). Raw cortisol values are shown in Plot D; log-transformed scores were used for analyses.

Participants in both conditions experienced similar changes in psychosocial stress during recovery with decreases in tension and anxiety and increases in well-being. There were no significant condition differences for tension *F*_(1, 91)_ = 1.56, *p* = 0.21, 95% CI [–0.29, 1.25], η^2^ = 0.02; anxiety *F*_(1, 88)_ = 0.004, *p* = 0.95, 95% CI [–1.01, 0.95], η^2^ = 0.00; or well-being *F*_(1, 91)_ = 0.33, *p* = 0.57, 95% CI [–0.49, 0.90], η^2^ = 0.004. Positive and negative affect were measured at baseline and follow up. While participants showed decreases in positive affect and increases in negative affect, there were no significant condition differences for either positive affect, *F*_(1, 91)_ = 2.50, *p* = 0.12, 95% CI [–4.78, 0.54], η^2^ = 0.03 or negative affect, *F*_(1, 91)_ = 0.053, *p* = 0.82, 95% CI [–2.20, 2.77], η^2^ = 0.01 when controlling for Facebook Intensity and gender (see Figures 2A–D for subjective stress markers).

### Effect of facebook use on physiological stress recovery

Preliminary analyses confirmed that all participants experienced physiological stress in response to the TSST (see Procedure). Participants showed a significant decrease in both blood pressure and heart rate from eight to 20 min post-stressor onset, indicating that recovery of heart rate and blood pressure occurred; however, there were no significant condition effects for systolic blood pressure *F*_(1, 91)_ = 0.16, *p* = 0.69, 95% CI [–3.60, 5.38], η^2^ = 0.002; diastolic blood pressure *F*_(1, 91)_ = 2.11, *p* = 0.15, 95% CI [–0.95, 6.13], η^2^ = 0.023, or heart rate *F*_(1, 91)_ = 1.05, *p* = 0.31, 95% CI [–1.65, 5.24], η^2^ = 0.012 when controlling for gender and Facebook Intensity score. Compared to control participants at baseline, participants in the Facebook use condition were more likely to report that using Facebook when stressed makes them feel *less* stressed (see Table 1). However, compared to the Facebook use condition (*M*_difference_ = –0.35, *SD* = 0.37), control participants (*M*_difference_ = –0.51, *SD* = 0.38) showed a significantly greater decrease in cortisol concentration from 20 to 45 min post-stressor onset when controlling for gender and Facebook Intensity score^2^, *F*_(1, 84)_ = 5.03, *p* = 0.03, 95% CI [0.21, 0.33], η^2^ = 0.06 (see Figures 2A–D).

**Figure 3:**
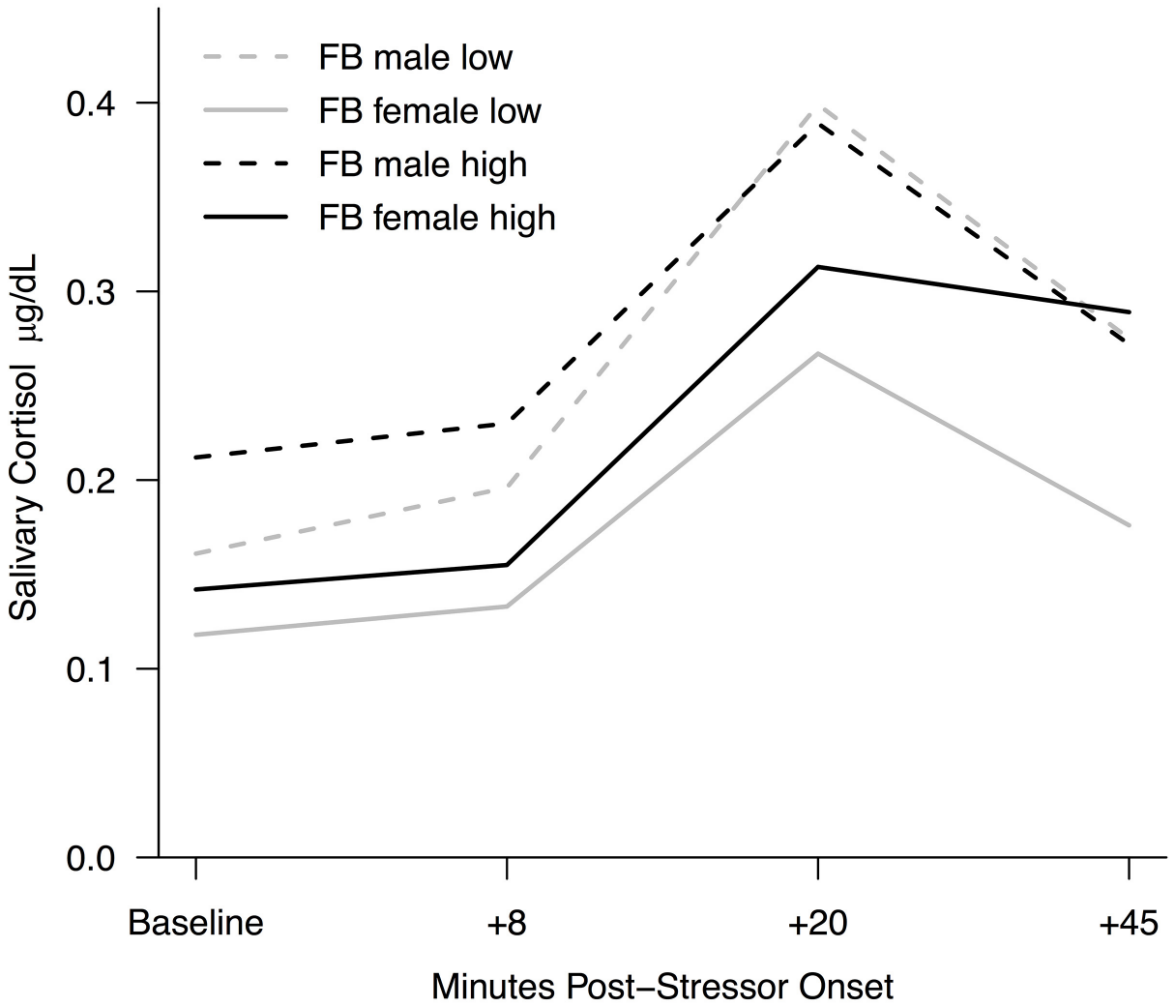
Salivary cortisol response to stress for the Facebook use condition by gender and high/low Facebook Intensity. Raw cortisol values are reported here; log-transformed values were used for analyses.

Secondary to assessing the effect of Facebook use on stress recovery, we explored how both investment in the website and gender may influence recovery. Although sample sizes did not allow for testing interaction effects, descriptively, females in the Facebook use condition who reported high levels of Facebook Intensity showed the smallest reduction in cortisol concentration during recovery (see Figure 3). That is, based only on descriptive mean differences, they remained the *most* stressed compared not only to control participants with high and low Facebook Intensity, and to males who used Facebook with high and low Facebook Intensity, but also compared to *females* who used Facebook with *low* levels of Facebook Intensity. A similar pattern was reflected when females (*M* = 2.70, *SD* = 0.94) were more likely than males (*M* = 2.16, *SD* = 0.98) to report that using Facebook when stressed makes them feel *more* stressed *t*_(90)_ = 2.65, *p* = 0.01, 95% CI [–0.93, –0.13], *d* = 0.46. This potential interaction between gender and investment in the website suggests that highly invested females may be more susceptible to social media-induced stress.

## Discussion

The present study provides the first objective evidence of how social media may affect stress. All participants experienced significant changes in subjective and physiological stress in response to an acute laboratory stressor; however, using Facebook inhibited physiological recovery. Specifically, participants who used Facebook during recovery showed sustained cortisol levels compared to control participants, suggesting that Facebook can *get under the skin*.

Given the mixed literature on Facebook use and well-being, several explanations for such findings exist. Social Self-preservation Theory poses that the social self-preservation system (including the HPA axis) tracks one's surroundings for threats to social status or social esteem. When a threat is present, both cortisol and negative self-related cognitions and emotions increase (Dickerson et al., 2004). Given that Facebook use has been associated with social status and social esteem (e.g., Ellison et al., 2011; Best et al., 2014), it is possible that Facebook use could be viewed as a threat to self-preservation and may induce similar physiological effects. In the present study, participants who immediately engaged with their own Facebook profile after experiencing an acute social stressor sustained significantly higher levels of objective stress compared to control participants (i.e., their recovery from stress was delayed). This extended stress response may reflect an additive effect of physiological and psychosocial arousal in response to threats to self-preservation (i.e., both the acute social stressor *and* Facebook were perceived as threats; therefore, participants who experienced both showed prolonged stress responses compared to those who only experienced the acute social stressor). This relationship may be particularly true for females highly invested in the site.

It is also possible that immediately engaging in a stimulating activity after experiencing acute stress may have reduced the likelihood of recovering from stress. All participants were at a heightened level of arousal at the beginning of recovery. Directly beginning another task (i.e., using Facebook) may have sustained higher levels of physiological arousal. Although using Facebook sustained cortisol, it had no effect on blood pressure or heart rate. This may have been due to the natural rapid recovery rate of these more acute markers (Linden et al., 1997). However, it is also possible that Facebook differently affects sympathetic-adrenomedullary (SAM) activity (blood pressure and heart rate) and HPA activity (cortisol). Future work may benefit from including additional and more precise measurement of the SAM system (e.g., salivary alpha-amylase).

Despite sustaining cortisol, Facebook did not sustain psychosocial stress. Those who used Facebook reported recovering as much as those in the control condition despite showing a sustained physiological stress response. This dissociation is consistent with findings demonstrating that the psychological experience of stress does not necessarily map on to a physiological response (e.g., Kirschbaum et al., 1995; Egloff et al., 2002; Inagaki and Eisenberger, 2016; Levi, 2016). In this specific context, this dissociation may aid in explaining mixed findings in the literature. For example, cross-sectional findings implicating associations between both Facebook use and enhanced well-being (Kim and Lee, 2011) *and* Facebook use and greater distress (Chen and Lee, 2013) may reflect a disconnection between what users experience and what they report. Although there were no significant effects of Facebook use on self-reported stress in this study, examining this relationship will remain important considering that the majority of participants in the Facebook use condition reported at baseline that using Facebook when stressed *reduces* stress.

While results suggest a link between social media use and stress, the implications for overall well-being are less clear. The context of acute stress provides valuable insight into how Facebook use may influence users, especially given that a significant portion of users report not only *wanting* to use the site when stressed, but that using it when stressed actually *reduces* stress. Subjective stress reduction may in fact occur; however, our findings highlight the importance of also considering physiological stress recovery. Given the known associations between stress and negative health and well-being outcomes (Herbert and Cohen, 1994), Facebook use after experiencing acute stress may not be recommended.

Despite these novel findings, limitations must be addressed. First, the majority of participants reported using the Facebook mobile app as the most common means of access. Use of the platform in such a context may have different implications for stress recovery, particularly given that mobile use implies a mobile environment (e.g., walking around in a public space). However, to best capture the effect of Facebook use on stress recovery in an experimental context, limiting platform access to a stationary laptop computer in a quiet room allowed us to rule out confounding factors. Future work assessing mobile use of the platform will require careful control of many external environmental factors. Second, in effort to provide some level of arousal for all participants, the control condition involved a stimulating, yet neutral activity. We did not assess if participants normally read magazines when feeling stressed. Third, use of a computer (vs. reading magazines) may have influenced physiological recovery. Future work may benefit from including a third condition involving complete rest. Fourth, participants may have accessed materials when using Facebook that could have differently affected arousal. As social media continues evolving, future work should consider how specific activities (e.g., posting photos, viewing videos, etc.) differently influence well-being. Similarly, the potentially variable impact of different text- and image-based platforms (e.g., Twitter, Instagram, and Snapchat) must also be considered. Finally, the broader social context of use must be acknowledged. For example, national and global-level events (e.g., the constant social media coverage of the contentious 2016 U.S. Presidential Election) may temporarily create an inherently stressful environment with otherwise undue consequences for well-being.

Although much work remains to be done, the present study provides the first experimental evidence that social media may in fact get under the skin. We show that when accounting for gender and investment in the website, using Facebook after facing an acute social stressor delays physiological stress recovery in terms of cortisol. That is, using Facebook when stressed sustains physiological stress. Future work must consider with greater precision, the influence of specific Facebook activities on both psychological and physiological well-being. Particular attention should be paid to user gender and investment in the website.

## Author contributions

HR developed the study concept. Both authors contributed to the study design. Testing and data collection were performed by HR. Immunoassay, data analyses, and interpretation were performed by HR under the supervision of JT. HR drafted the manuscript, and JT provided critical revisions. Both authors approved the final version of the manuscript for submission.

### Conflict of interest statement

The authors declare that the research was conducted in the absence of any commercial or financial relationships that could be construed as a potential conflict of interest.

## Footnotes

^1^Note that the first saliva sample was not collected until ~20 min after each participant arrived. Thus, participants had refrained from these activities for at minimum 50 min before saliva collection began.

^2^Controlling for BMI and use of hormonal contraceptives did not change significant results.
